# NEDA-3 using cladribine for multiple sclerosis: effectiveness data from a Norwegian hospital

**DOI:** 10.3389/fneur.2025.1698334

**Published:** 2026-01-05

**Authors:** Nora Berg Bjørnevoll, Karl Bjørnar Alstadhaug

**Affiliations:** 1 Faculty of Health Sciences at UiT – The Arctic University of Norway, Tromsø, Norway; 2 Department of Neurology, Nordland Hospital, Bodø, Norway; 3 Institute of Clinical Medicine, The Arctic University of Norway, Tromsø, Norway

**Keywords:** multiple sclerois, cladribine, NEDA-3, effectivness, survival analaysis

## Abstract

**Background:**

Cladribine is an immunomodulatory agent used in the treatment of relapsing–remitting multiple sclerosis (MS) and, in some countries, for active progressive forms of MS. However, relatively few clinical observational studies have evaluated its effect.

**Materials and methods:**

This retrospective cohort study included all patients with multiple sclerosis who were treated with cladribine at Nordland Hospital between April 2018 and September 2024. We aimed to evaluate the effectiveness of cladribine by assessing No Evidence of Disease Activity (NEDA)-3 over time.

**Results:**

A total of 60 patients (62% women), of whom 58 had relapsing–remitting multiple sclerosis, 1 had radiologically isolated syndrome, and 1 had secondary progressive disease and received at least 1 cyclic treatment. The mean age at the initiation of cladribine was 38.5 ± 11.0 years, and the mean Expanded Disability Status Scale (EDSS) was 2.1 ± 1.3. Two-thirds (65%) of the patients had received other disease-modifying treatment before cladribine. During the study period, eight patients switched or discontinued treatment, seven due to disease activity, two due to self-financed autologous stem cell treatment, one due to a drug overdose, and one for an unclear reason. Compared to patients with evidence of disease activity (EDA), those achieving NEDA-3 after 2 years were older at MS onset (*p* = 0.046), and a larger proportion were treatment-naïve at the start of cladribine (42% vs. 11%, *p* = 0.03). NEDA-3 was achieved by 36 (60%) patients at 1 year, 24 (40%) at 2 years, and 20 (33%) at 3 years. Mild to moderate adverse events were observed or reported in 13 (21.7%) patients; 1 developed lymphocytopenia grade 3, and 1 developed herpes zoster.

**Conclusion:**

One in three patients achieved NEDA-3 3 years after starting cladribine, with the best outcomes observed in treatment-naïve patients, while its favorable safety profile and convenient dosing support long-term adherence and disease stability.

## Introduction

Multiple sclerosis (MS) is a chronic, autoimmune disease characterized by inflammatory lesions and demyelination in the central nervous system. It is one of the most common causes of neurological impairment in young adults and is typically diagnosed between the ages of 20 and 40 years (1, 2). Pathogenesis is not fully understood, but it is believed to be due to a genetic predisposition in combination with adverse environmental factors. Aberrant immunological response to the Epstein–Barr virus is likely an important mechanism (3). Autoreactive T and B cells contribute to the formation of inflammation that leads to damage to myelin, oligodendrocytes, and nerve fibers (1–3). Relapsing–remitting MS (RRMS) is the most common form of MS. It is characterized by episodes of inflammation and neurological symptoms followed by periods of partial or complete remission (4). In progressive MS, neurodegeneration becomes more prevalent. Both inflammation and neurodegeneration lead to functional impairment (2). The Expanded Disability Status Scale (EDSS) is used to assess disease progression.

In 2017, cladribine, a nucleoside analog of deoxyadenosine initially developed for the treatment of various types of cancer, was approved for use in RRMS in Europe (5). In April 2018, the drug was also approved for use in Norway (6). According to the 2022 national guidelines (7), high-efficacy drugs, including cladribine, could be offered to patients with newly diagnosed RRMS. In Norway, cladribine is not offered to patients with active secondary progressive MS, unlike in some countries (8).

Cladribine reduces lymphocyte numbers through programmed cell death (apoptosis) rather than through cell lysis. This gradual elimination of lymphocytes over several months is considered advantageous for the safety profile of cladribine, as it prevents massive cytokine release that can lead to the reactivation of demyelinated lesions (9, 10). Cladribine is given over a period of 2 years, with a cumulative dose of 3.5 mg/kg body weight over a period of 2 weeks per year (11).

The use of cladribine in MS is based on the findings of Cladribine Tablets Treating Multiple Sclerosis Orally (CLARITY) studies. A randomized controlled trial with 1,184 of 1,326 patients who completed a 96-week study showed a significant reduction in the annual relapse rate compared with placebo (relative risk reduction of 57.6%), as well as a reduced risk of increased functional loss at 3 and 6 months with the use of cladribine tablets 3.5 mg/kg (11). To evaluate the effect of an MS drug in routine clinical practice, No Evidence of Disease Activity (NEDA) is often used as a measure (see under the Methods section). In the CLARITY Extension study, the current treatment regimen (n = 98) had 39.8% NEDA-3 after 1 year and 29.6% after 2 years (12). Beyond the CLARITY studies, there are a limited number of studies evaluating the efficacy and safety profile of cladribine for the treatment of MS (2, 8, 13–17).

Studies indicate that cladribine has a slower onset of action and generally lower efficacy than other high-efficacy therapies (HETs), including off-label rituximab (18, 19). However, a recent registry study from Argentina, evaluating NEDA-3 among 462 patients receiving different HETs, found no significant differences between cladribine and monoclonal antibodies (13). Moreover, some studies indicate that it is safe and effective to switch from natalizumab to cladribine (14), whereas others have found an increased risk of disease activity (15). In Norwegian clinical practice, the choice of HET is made on an individual basis, considering patients’ preferences, comorbidities, administration methods, and human polyomavirus 2 (JCV) status. Regional differences in treatment practice have been evident in Norway, with off-label rituximab being the preferred HET in the southwest at Haukeland University Hospital, while on-label cladribine has primarily been used in the southeast at Oslo University Hospital (19).

In this study, we aimed to assess all patients treated with cladribine over a 6-year period at Nordland Hospital, to evaluate NEDA-3 over time, and to examine the clinical rationale for initiating cladribine compared with other HETs.

## Materials and methods

This was a retrospective cohort study conducted at Nordland Hospital in Bodø, Norway. All patients with MS who were treated with cladribine in the Neurological Department between April 2018 and September 2024 were included in the study. Data collection was conducted between October and December 2024. Approximately 660 patients with MS were living in Nordland during this period (16), and data from the Norwegian MS registry and local administrative data show that, of the 463 patients who were regularly followed up, 364 were treated with immunomodulatory drugs. Of these, 121 were treated with rituximab (33%), 60 with cladribine (17%), 50 with dimethyl fumarate (14%), 22 with teriflunomide (6.0%), 22 with fingolimod (6.0%), 11 with glatiramer acetate (3.0%), 11 with natalizumab (3.0%), 10 with alemtuzumab (3%), and 17 with other drugs (5%), including ofatumumab, interferon beta-1b, interferon beta-1a, ozanimod, peginterferon beta-1a, and ocrelizumab. In addition, 32 (8%) had received hematopoietic stem cell transplantation, of which 4 were treated in Norway and 28 were treated abroad, and a total of 99 patients were registered without any treatment.

### The study subjects

All patients diagnosed with RRMS according to the McDonald criteria (20) and who had received at least one dose of cladribine between 2018 and 2024 were included in the study. Patients were scheduled for follow-up visits with an MS nurse at 3 months and with a neurologist at 6 months after the first treatment cycle and annually thereafter. At least one follow-up visit was required for participation in this study. Standard treatment was given according to the Common Catalog text, with a cumulative dose of 3.5 mg/kg over 2 years. Each treatment cycle was administered over 4–5 days for 2 weeks per year for 2 years.

### Data collection

Patients were identified via the hospital’s electronic medical record system (DIPS, ASA, Oslo, Norway). The variables in the dataset were obtained manually through a thorough review of the records of each individual patient. Missing EDSS scores were retrospectively calculated. For patients who moved during the follow-up period or were later treated at other hospitals, medical records were obtained from the current hospital. Information on adverse events was primarily collected during scheduled follow-up visits, and some patients reported events independently between visits.

### Objectives of the study

The primary objective of this study was to evaluate NEDA-3. NEDA-3 is achieved when, at evaluation, there is (1) the absence of clinical MS relapse, (2) the absence of disability progression corresponding to ≥ 0.5-point increase in EDSS, and (3) the absence of gadolinium-enhancing lesions or new T2-hyperintense lesions on MRI. NEDA-3 was evaluated from the time of treatment initiation rather than from a baseline MRI. Secondary objectives included evaluating the safety profile of cladribine and comparing patients who achieved NEDA-3 with those exhibiting Evidence of Disease Activity (EDA).

### Statistical analyses

SPSS (IBM Corporation, Armonk, NY, version 29) was used to perform statistical analyses. Survival curves were used to estimate and demonstrate NEDA-3 over time and to compare the effect of cladribine on women and men. Student’s t-test, the Mann–Whitney U-test, and the chi-squared test were used to compare demographic data. The threshold for statistical significance was set at a *p*-value of < 0.05.

### Ethics and recommendations

The study was conducted in accordance with the ethical principles of medical research as outlined in the Declaration of Helsinki. The study was considered a quality assurance project and was recommended by the clinic management and the hospital’s data protection officer (PVO reg. 397).

## Results

### Patient characteristics and clinical data

During the given period, 60 patients had been treated with at least the first cyclic treatment (1 year, corresponding to half the recommended cumulative dose of 3.5 mg/kg body weight). Of these, 58 were diagnosed with RRMS, 1 with radiologically isolated syndrome (RIS), and 1 with secondary progressive MS. The mean age at inclusion was 42.7 ± 10.62 years, and the majority were women (62%). The mean age at disease onset and at diagnosis was 32.6 ± 9.4 years and 35.0 ± 10.1 years, respectively. The mean EDSS at diagnosis was 1.6 ± 1.2, and the mean time from onset to diagnosis was 2.1 ± 3.9 years. Five patients were refugees, four from Ukraine and one from Syria. A total of 25 patients had comorbid psychiatric disorders, and 15 of them had migraine in addition to the MS diagnosis (Table 4).

There was a significant difference between women and men in terms of leukocytes (*p* = 0.005) and total protein (<0.001) in cerebrospinal fluid (CSF) at diagnosis. A mean value of vitamin D at diagnosis of 60.1 ± 31.7 nmoL/L was also noted, of which 46.9% (15/32) had a value below 50 nmoL/L. Table 1 summarizes demographic and diagnostic data.

**Table 1:** Demographic and diagnostic data for the MS population.

	Women (*n* = 37)	Men (*n* = 23)	Total (*n* = 60)	*p*-value
Debut				
Age at MS onset (years), mean ± SD	32.59 ± 9.2	32.48 ± 10.02	32.55 ± 9.44	0.96
Diagnose				
Age at diagnosis (years), mean ± SD	35.76 ± 10.36	33.65 ± 9.63	34.95 ± 10.06	0.44
EDSS at diagnosis, mean ± SD (*n* evaluated)	1.65 *±* 1.37 (34)	1.55 ± 1.01 (20)	1.61 ± 1.24 (54)	0.78
Number of relapses at diagnosis, mean ± SD	1.46 ± 0.99	1.57 ± 1.12	1.50 ± 1.03	0.70
Leukocytes in CSF, mean ± SD (*n* evaluated)	6.82 ± 5.04 (33)	15.05 ± 14.86 (19)	9.83 ± 10.49 (52)	0.005
Total protein in CSF at first evaluation, mean ± SD (*n* evaluated)	0.34 ± 0.09 (31)	0.47 ± 0.11 (19)	0.39 ± 0.12 (50)	<0.001
Vitamin D, nmol/l, mean ± SD (*n* evaluated)	60.45 ± 35.2 (20)	59.42 ± 26.21 (12)	60.1 ± 31.7 (32)	0.93
Time from diagnosis to first disease-modifying treatment (months), median (IQR)	0.83 (3.9)	0.65 (1.46)	0.77 (1.8)	0.26

### Treatment with cladribine

The mean age and EDSS at the start of cladribine were 38.5 ± 11.0 years and 2.1 ± 1.3 years, respectively. Among women, the mean time from diagnosis to the start of disease-modifying therapy (DMT) and to the initiation of cladribine was 21.1 ± 56.4 and 62.5 ± 91.0 months, respectively; for men, the corresponding times were 2.74 ± 5.83 and 38.5 ± 54.3 months.

Two-thirds of the patients had used other DMT before starting cladribine (Table 2). Before starting cladribine, they had been treated with dimethyl fumarate (*n* = 15), teriflunomide (*n* = 9), fingolimod (*n* = 4), glatiramer acetate (*n* = 4), interferon (*n* = 3), rituximab (*n* = 2), and natalizumab (*n* = 2). The primary reasons for switching to cladribine were treatment failure (49%) and adverse effects (46%). Additionally, one patient switched to make it easier to plan a future pregnancy, and one requested a change based on personal preference. The choice of cladribine as subsequent therapy was based on its favorable safety profile (25%), the treating neurologist’s consideration of it as the best or most convenient therapeutic option (50%), patient preference (11.7%), or a desire to conceive (6.7%). Two patients switched to cladribine due to participation in a randomized clinical trial (RAM-MS). None of the patients experienced a rebound upon switching to cladribine.

**Table 2:** Treatment with cladribine.

	Women (*n* = 37)	Men (*n* = 23)	Total (*n* = 60)	*p*-value
Before treatment				
Patients with prior *DMT use, *n* (%)	23 (62.2)	16 (69.6)	39 (65)	0.56
Time from diagnosis to 1. treatment with cladribine (months), median (IQR)	18.9 (74.6)	15.5 (41.9)	17 (69.4)	0.42
**EDSS, mean ± SD	2.074 ± 1.35	2.03 ± 1.31	2.06 ± 1.32	0.90
EDSS <3.0, *n* (%)	22 (64.7)	15 (78.9)	37 (69.8)	0.28
EDSS ≥ 3.0, *n* (%)	12 (35.3)	4 (21.1)	16 (30.2)	0.28
Treatment				
Age (years) at first dose, mean. ± SD	39.70 ± 11.45	35.65 ± 10.08	38.53 ± 10.96	0.30
Reported side effects, *n* (%)	12 (32.4)	1 (4.3)	13 (21.7)	0.01
Leukopenia, *n* (%)	4 (10.8)	3 (13.0)	7 (11.7)	0.71
***NEDA-3 after 1 year, *n* (%)	22 (59.5)	14 (60.9)	36 (60)	0.51
NEDA-3 after 2 years, *n* (%)	15 (40.5)	9 (39.1)	24 (40)	0.78
NEDA-3 after 3 years, *n* (%)	11 (29.7)	9 (39.1)	20 (33.3)	0.14

*DMT, disease-modifying therapy; **EDSS, Expanded Disability Status Scale; ***NEDA-3, No Evidence of Disease Activity.

Eight patients switched or discontinued the cladribine treatment, five after the first cycle, two due to disease activity, two due to self-financed autologous stem cell transplantation, and one who died of a drug overdose. After completing the 2-year cladribine course, three patients initiated another DMT: two because of disease activity (one switched to rituximab and one to ofatumumab) and one switched to rituximab for an unclear reason.

Adverse events were reported by 13 (21.7%) patients and were mild to moderate in severity. These included hair loss (*n* = 4), fatigue (*n* = 2), headache (*n* = 2), rash (*n* = 2), abdominal pain (*n* = 1), muscle cramps (*n* = 1), and herpes zoster (*n* = 1). Seven patients developed leukopenia; in six cases, this was transient, whereas lymphopenia was observed in only one patient (grade 3, 200–499 × 10⁶/L).

### Primary outcome

**Figure 1:**
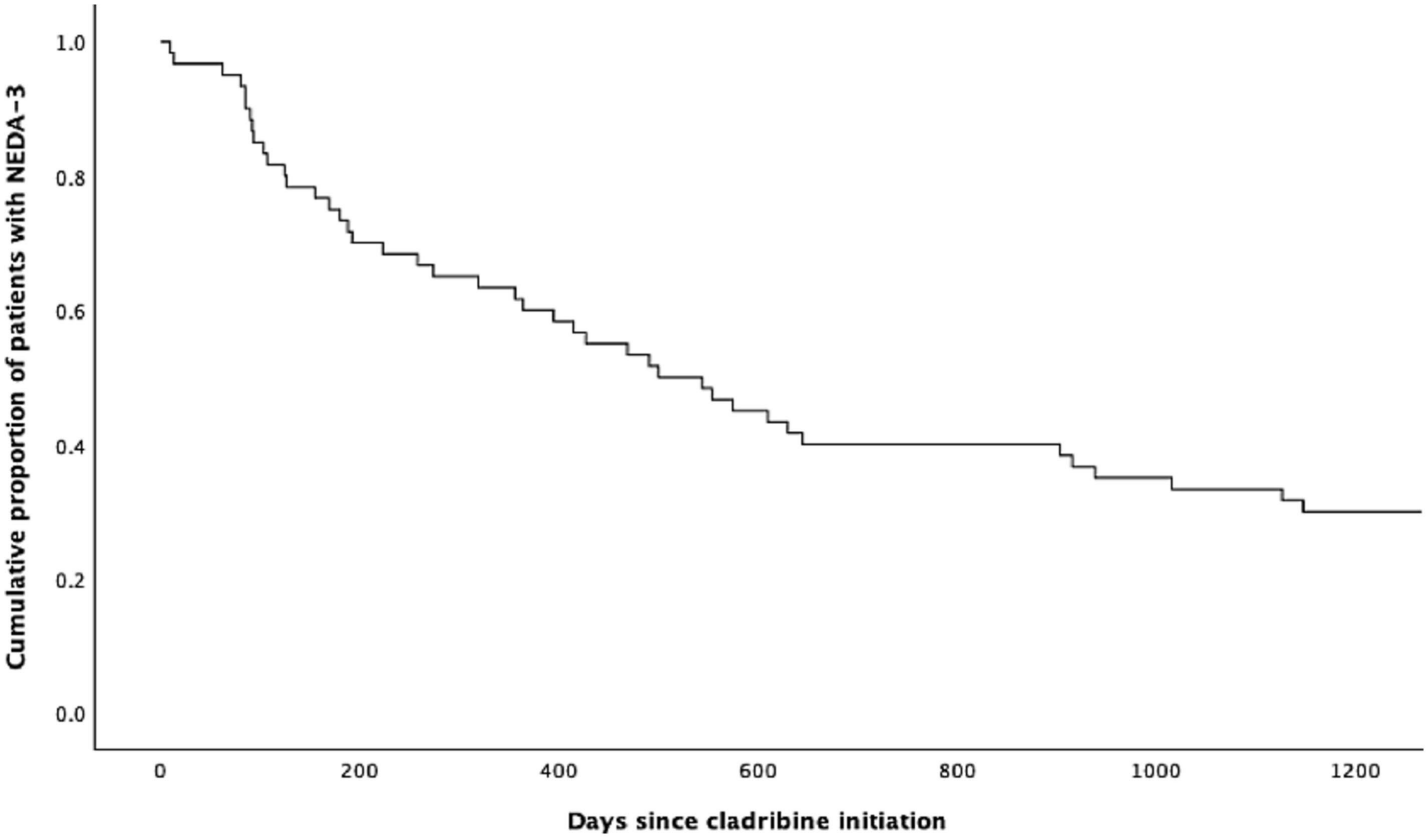
The Kaplan–Meier curve for patients with multiple sclerosis (*n* = 60) achieving NEDA-3 following cladribine treatment.

Table 2 shows the NEDA-3 status over time. NEDA-3 after 1, 2, and 3 years was 60% (36/60), 40% (24/60), and 33.3% (20/60), respectively, as demonstrated graphically in Figure 1. Table 3 shows the differences between patients who achieved NEDA-3 and those with EDA after 2 years. MRI changes were the primary reason for the loss of NEDA (18/26), and the majority of patients lost NEDA within the first 6 months (13/26) (not shown in tables). Patients who achieved NEDA-3 were significantly older at disease onset (*p* = 0.046), and a larger proportion were treatment-naïve compared to patients with EDA. Notably, EDSS at diagnosis was lower in those who achieved NEDA-3, although this difference was not statistically significant.

**Table 3:** NEDA-3 vs. EDA after 2 years.

	*EDA (*n* = 18)	**NEDA-3 (*n* = 24)	*p*-value
Age at MS- debut (years), mean *±* SD	28.39 *±* 6.73	33.83 *±* 9.56	0.046
Age at diagnosis (years), mean *±* SD	31.5 *±* 9.02	35.92 *±* 9.79	0.14
Age at first dose (years) cladribine, mean. *±* SD	34.2 *±* 9.03	39.96 *±* 10.97	0.08
**EDSS at the time of diagnosis, mean *±* SD (*n* evaluated)	1.47 *±* 1.45 (16)	1.63 *±* 1.21 (23)	0.43
EDSS before first cladribine treatment, mean *±* SD (*n* evaluated)	1.7 *±* 1.47 (15)	2.24 *±* 1.45 (21)	0.28
Time from diagnosis to first treatment (months), median (IQR)	0.65 (1.6)	0.8 (3.2)	0.44
Time from diagnosis to first dose of cladribine (months), median (IQR)	35.2 (105)	13.2 (46.9)	0.18
Cladribine as first-line treatment, *n* (%)	2 (5)	10 (23.5)	0.03

*EDA, Evidence of Disease Activity; **NEDA-3, No Evidence of Disease Activity.

**Figure 2:**
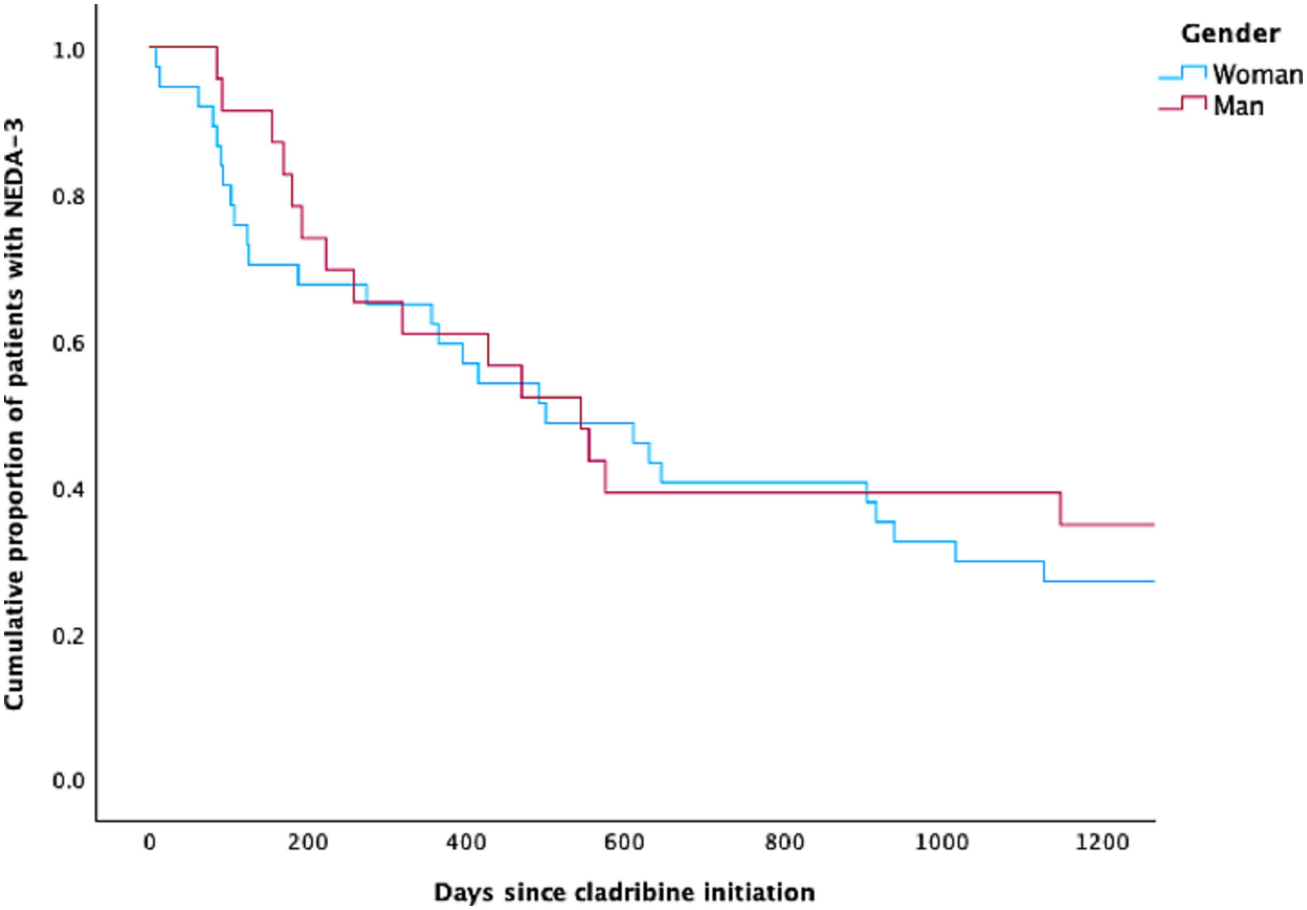
The Kaplan–Meier curve for women and men with multiple sclerosis achieving NEDA-3 following cladribine treatment.

**Table 4:** Status 2024.

	Women (*n* = 37)	Men (*n* = 23)	Total (*n* = 60)	*p*-value
Status 2024				
Age (years), mean *±* SD	44.24 *±* 10.58	40.22 *±* 0.43	42.7 *±* 10.62	0.16
Smoking, n_yes_/n_earlier_ (%)	17/35 (48.6)	11/21 (52.4)	28/56 (50.0)	0.75
Psychiatric diagnosis, *n* (%)	16 (43.2)	9 (39.1)	25 (41.7)	0.75
Migraine, *n* (%)	11 (29.7)	4 (17.4)	15 (25)	0.28

## Discussion

The efficacy of cladribine, with an NEDA-3 of 40% after 2 years, is consistent with findings from previous studies. In the CLARITY study, 44% of patients achieved NEDA-3 (17), while the CLARITY Extension study reported 29.6% (12). A Danish population-based longitudinal cohort study found a prevalence of 49% after 24 months among 113 evaluated patients (21). In a recent Norwegian study comparing the efficacy of rituximab and cladribine, 15% of patients treated with cladribine (*n* = 40) achieved NEDA-3 after 4.4 years compared with 57% of those treated with rituximab (*n* = 56) (19).

Compared with the study from Denmark, the time from diagnosis to initiation of cladribine was shorter in our study (4.5 vs. 7.7 years). In our study, NEDA-3 was calculated from treatment initiation, unlike the CLARITY studies and the Danish study, where it was calculated from a new MRI scan (re-baseline MRI). This methodological difference could potentially have resulted in MRI lesions being carried over and an incorrect loss of NEDA-3 status in the first year, resulting in an underestimation of the treatment effect in our study. On the other hand, new lesions during the initial period after the initiation of high-efficacy (19) induction therapy (22) are a well-known phenomenon and should, of course, be taken into account when evaluating effectiveness.

A higher proportion of patients in our study were treatment-naïve compared to the patients in the abovementioned studies, except for the CLARITY Extension study, where 82% were first-time users. A recent study from Italy observed the greatest reduction in annual relapse rate among treatment-naïve patients, whereas a higher number of prior treatments was associated with a lower likelihood of maintaining NEDA-3 status and a higher risk of clinical relapse (23). In recent years, clinical practice in MS treatment has shifted toward more liberal and earlier use of high-potency therapies. This may result in a higher proportion of patients starting cladribine having less aggressive MS than those in the pivotal studies. Significantly better 2-year outcomes have been reported in two observational studies: 74.9% NEDA-3 in an Italian cohort (24) and 75% in a Kuwaiti cohort (25). This is probably largely because the first 6 months after starting cladribine were not included. In the Italian study, 7 of 114 patients (6%) experienced a clinical relapse, and 36 (32%) showed MRI changes within the first 6 months after starting treatment. The study authors concluded that disease activity in the first 6 months did not increase the risk of greater activity on subsequent follow-up and that some degree of initial disease activity may be tolerated until the second treatment cycle is completed.

All studies, including ours, show that cladribine has a favorable safety profile. A moderate number of patients experienced mild to moderate side effects, which is consistent with findings from other clinical studies. The favorable safety profile was also one of the main reasons why patients in our cohort chose to initiate cladribine treatment. Only one of our patients developed grade 3 lymphocytopenia, but this persisted as grade 2. In the CLARITY Extension, 5 out of 98 developed grade 3 lymphocytopenia; however, all reestablished lymphocyte counts corresponded to grades 0–1 within a mean of 41 days (26). Reported across studies, the main reason for switching or discontinuing cladribine treatment has been disease activity.

An interesting observation in the CLARINET-MS study was that, after 60 months, the estimate for the probability of experiencing disability progression was 63.7%, while the corresponding probability of receiving a treatment change was only 28.1%. This was interpreted in the study as a potential indication that treatment decisions were not solely driven by measurable disease progression. Instead, it suggests that other factors, such as physician uncertainty about cladribine’s long-term effects, the absence of relapses, or a lack of clear treatment guidelines, may have influenced the decision to continue without switching therapy (27).

Our study shows that treatment with cladribine may be characterized by a pragmatic approach. We previously assessed the effectiveness of natalizumab in our MS cohort, which showed a prevalence of 50% for NEDA-3 after 3 years (28). Despite this and the fact that rituximab also has a better and earlier onset of effect (19, 29), we have chosen to use cladribine where it has been considered most appropriate in relation to the probability of good compliance with the treatment. The advantage of treating with cladribine is that it is simple, short-term, and easy to monitor. A high proportion of patients in our study had a mental disorder, and some were refugees.

The limitations of this study include the small cohort size and its retrospective design. Not all patients underwent a new MRI before starting cladribine, and clinical and radiological assessments were not always ideally timed, unlike in a prospective study. Moreover, part of the study was conducted during the COVID-19 pandemic, which led to several of the patients having their clinical follow-up and MRI examination canceled or postponed. Some consultations during this period were conducted via video or telephone, making EDSS scoring impossible. There was also some missing information in some medical records, including EDSS evaluations. These scores were therefore calculated retrospectively based on documented findings from the clinical examinations. Furthermore, treatment efficacy was evaluated using NEDA-3, which is no longer considered fully representative of disease control. Assessment using NEDA-4, which additionally accounts for annual brain volume loss of <0.4%, provides a broader assessment of treatment efficacy (30). The strengths of this study include regular 6-monthly medical follow-up of MS patients, annual MRI examinations, regular contact with an MS nurse, full access to data, and thorough review of medical records.

In conclusion, cladribine is an effective and well-tolerated therapy for RRMS that promotes good adherence. One in three patients achieved NEDA-3 3 years after treatment initiation, with the best outcomes observed among treatment-naïve patients. With its favorable safety profile, convenient administration, and suitability for patients in whom adherence or comorbidities may be a concern, cladribine represents a valuable and pragmatic option in individualized MS treatment.

## Data Availability

The original contributions presented in the study are included in the article/supplementary material, further inquiries can be directed to the corresponding author.
